# A Novel Formulation Based on Resveratrol and Water Extracts from *Equisetum arvense*, *Crataegus curvisepala*, *Vitex agnus-castus*, and *Glycine max* Inhibits the Gene Expression of Inflammatory and Osteoclastogenic Biomarkers on C2C12 Cells Exposed to Oxidative Stress

**DOI:** 10.3390/foods14050896

**Published:** 2025-03-06

**Authors:** Simonetta Cristina Di Simone, Alessandra Acquaviva, Maria Loreta Libero, Nilofar Nilofar, Fatma Tunali, Mariachiara Gabriele, Angelica Pia Centulio, Gianluca Genovesi, Davide Ciaramellano, Lucia Recinella, Sheila Leone, Luigi Brunetti, Gokhan Zengin, Giustino Orlando, Luigi Menghini, Annalisa Chiavaroli, Claudio Ferrante

**Affiliations:** 1 Botanic Garden “Giardino dei Semplici”, Department of Pharmacy, “G. d’Annunzio” University, 66100 Chieti, Italy; simonetta.disimone@unich.it (S.C.D.S.); alessandra.acquaviva@unich.it (A.A.); maria.libero@unich.it (M.L.L.); nilofar.nilofar@unich.it (N.N.); fatma.tunali@unich.it (F.T.); mariachiara.gabriele@studenti.unich.it (M.G.); angelicapia.centulio@phd.unich.it (A.P.C.); gianluca.genovesi@phd.unich.it (G.G.); d.ciaramellano@unidav.it (D.C.); lucia.recinella@unich.it (L.R.); sheila.leone@unich.it (S.L.); luigi.brunetti@unich.it (L.B.); giustino.orlando@unich.it (G.O.); luigi.menghini@unich.it (L.M.); claudio.ferrante@unich.it (C.F.); 2 Physiology and Biochemistry Laboratory, Department of Biology, Science Faculty, Selcuk University, Konya 42130, Turkey; gokhanzengin@selcuk.edu.tr

**Keywords:** *Equisetum arvense*, *Crataegus curvisepala*, *Vitex agnus-castus*, *Glycine max*, phenolic compounds, menopause, oxidative stress

## Abstract

Medicinal plants and natural compounds have been considered alternative therapeutic options for counteracting postmenopausal disorders thanks to their different concomitant effects, including antioxidant and anti-inflammatory properties and the regulation of hormone activity. It is important to highlight that the efficacy of medicinal plants and natural compounds increases when used in combination, thus making the development of herbal formulations rational. Therefore, the present study aimed to evaluate the phytochemical and pharmacological properties of an innovative formulation consisting of resveratrol and water extracts from *Equisetum arvense*, *Crateagus curvisepala*, *Vitex agnus-castus*, and *Glycine max*. The phenolic composition and radical scavenger properties were evaluated using chromatographic and colorimetric (ABTS) methods, whilst the limits of biocompatibility were assessed through allelopathy, the *Artemia salina* (brine shrimp) lethality test, and *Daphnia magna* cardiotoxicity assay. The protective effects were evaluated on C2C12 cell lines exposed to the pro-oxidant stimulus, which consisted of hydrogen peroxide. The gene expression of estrogen 1 (ESR1, also known as ERα) and prolactin (PRLR) receptors, interleukin 6 (IL-6), tumor necrosis factor α (TNFα), and receptor activator of nuclear factor kappa-Β ligand (RANKL) was measured. The results of the phytochemical analysis showed that the main phytochemicals were hydroxycinnamic and phenolic acids, in particular coumaric acid (7.53 µg/mL) and rosmarinic acid (6.91 µg/mL), respectively. This could explain the radical scavenger effect observed from the 2,2-azinobis (3-ethylbenzothiazoline-6-sulfonic acid) (ABTS) assay. According to the ecotoxicological models’ results, the formulation was revealed to be non-toxic, with a LC_50_ value > 1 mg/mL. Therefore, a biocompatible concentration range (200–1000 µg/mL) was used in C2C12 cells, where the formulation blunted the hydrogen peroxide-induced upregulation of TNFα, IL-6, RANKL, ESR1, and PRLR. Overall, the results of this study corroborate the use of the formulation for facing the oxidative stress and inflammation, which forms the basis of the osteoclastogenic process.

## 1. Introduction

**Figure 1:**
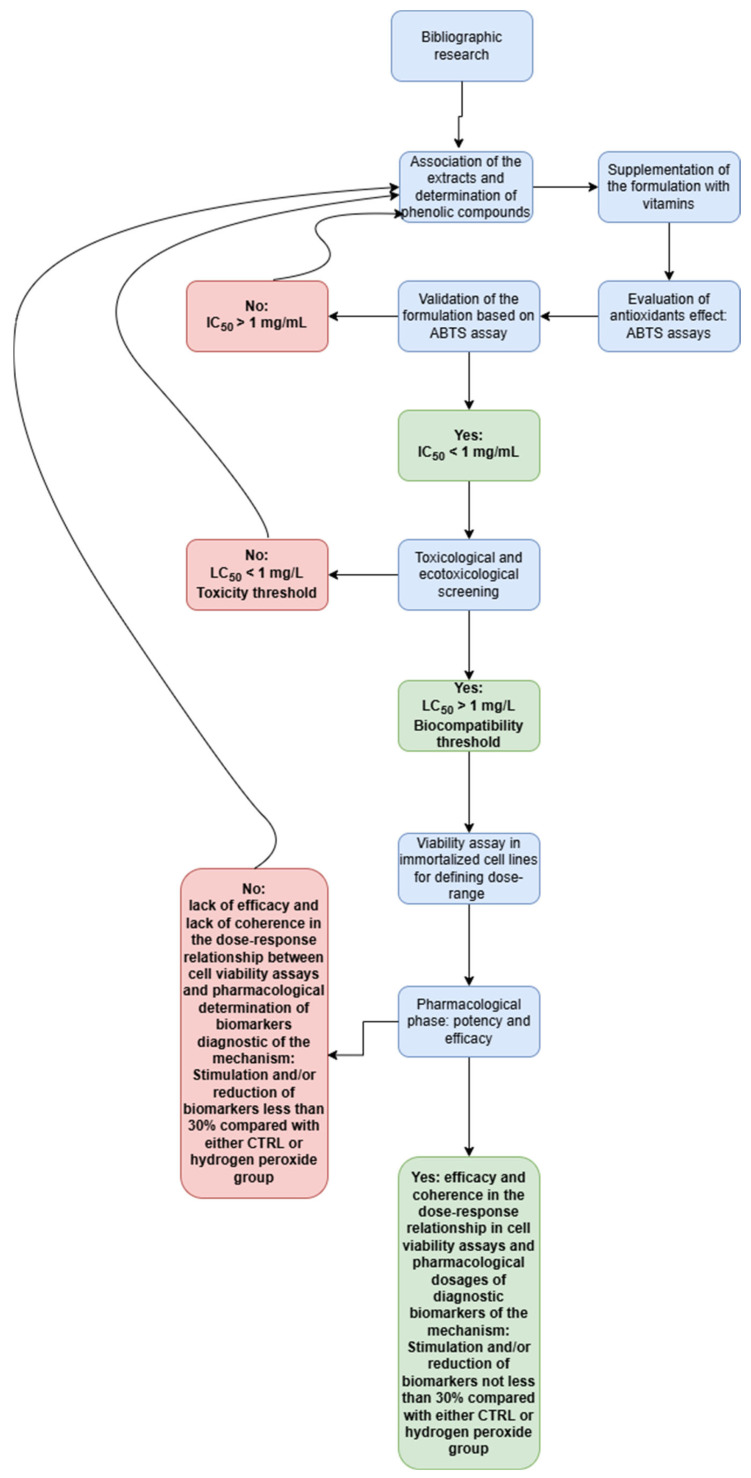
Flowchart of the study.

Oxidative stress has long been considered an imbalance in pro-oxidant and antioxidant homeostasis that leads to the overproduction and accumulation of reactive oxygen (ROS) and nitrogen (RNS) species, thus driving lipoperoxidation reactions and tissue damage [1]. During aging, oxidative stress has been demonstrated to play a pivotal role in disrupting the homeostasis between bone resorption and osteogenesis, both in men and women with a concomitant reduction in bone density and increase in the levels of oxidative stress biomarkers [2]. A major incidence of this process of imbalance is present in women in the postmenopausal phase [3], which is also characterized by the onset of estrogen reduction-related psychophysical and vasomotor symptoms, including hot flashes and oversensitivity to cold [4,5]. The reduction in estrogen levels is itself related to oxidative stress and reduced osteoblast activity in women [6]. First-line treatment of postmenopausal conditions includes different therapeutic options: estrogens for controlling hot flashes and preventing osteoporosis [7,8] and anabolic and anti-resorptive agents, particularly bisphosphonates, for facing osteoporosis [9]. Despite effectiveness, serious adverse reactions, including thromboembolic effects for estrogenic therapy [10], osteonecrosis of the mandible and gastrointestinal diseases related to the use of bisphosphonates [11], may occur. Alternative therapeutic options for counteracting postmenopausal disorders could derive from the use of medicinal plants, which, although characterized by minor efficacy compared with synthetic drugs, are often safer to use, due to a lower incidence of severe adverse reactions [12]. In this context, medicinal plants and natural compounds such as *Equisetum arvense*, *Crataegus* species, *Vitex agnus-castus*, *Glycine max*, *Polygonum cuspidatum*, and resveratrol have been found to be effective in promoting osteogenesis and reducing bone resorption, but also in managing hot flashes [13,14,15,16,17,18]. In this regard, it is imperative to highlight that a clinical trial conducted on postmenopausal women demonstrated the efficacy of *V. agnus-castus* against vasomotor symptoms, especially in women that had contraindications to the use of hormone therapy [13]. Additionally, the association of *E. arvense* with *G. max* extracts improved bone homeostasis in aged female rats [18]. The efficacy of the abovementioned medicinal plants may be related to their different concomitant effects, including antioxidant and anti-inflammatory activities and the regulation of hormone activity [9,17]. As well as medicinal plants, the use of vitamins has also been reported to improve women’s health in the postmenopausal phase. Indeed, vitamin K2 supplementation was shown to improve bone mineral density, whilst vitamin D3 has a well-known role in increasing intestinal calcium and phosphate absorption and their mobilization from the skeleton, in order to keep their levels constant [19,20]. It is important to highlight that the efficacy of herbal extracts and micronutrients seems to increase when used in combination, in both improving bone homeostasis and reducing the burden of inflammation and oxidative stress [21]. In the case of herbal extracts, those prepared via traditional infusions or decoctions have the advantage of being more biocompatible because of the use of water as the extraction solvent, thus being safer for long-term use [22,23]. The use of water extracts could be a novel strategy for developing sustainable products as well, with the advantage of boosting local botanical chain productions in the event that the plant material comes from medicinal plants traditionally used by folk populations to deal with chronic conditions, including osteoporosis, as in the case of the *Crataegus* species [15]. Therefore, the aim of the present study was to evaluate the pharmacological effect of an innovative natural formula formed from vitamin K2, vitamin D3, resveratrol from roots of *P. cuspidatum*, and water extracts from *E. arvense*, *C. curvisepala*, *V. agnus-castus*, and *G. max.* In this context, the study was conducted in vitro on C2C12 cell lines challenged with hydrogen peroxide pro-oxidant stimulus [21]. In this context, the gene expression of estrogen (ESR1, also known as ERα) and prolactin (PRLR) receptors with stimulating and inhibiting effects on bone formation was evaluated, respectively [24,25], as well as pro-inflammatory biomarkers including interleukin-6 (IL-6) and the tumor necrosis factor α (TNFα) that have long been involved in bone turnover through the stimulation of osteoclast function [26]. Additionally, the gene expression of a specific factor involved in bone remodeling, namely the receptor activator of the nuclear factor kappa-Β ligand (RANKL) was measured as well [27]. Finally, on the basis of the phytochemical composition of the natural formula evaluated through liquid chromatography analyses [28], a bioinformatics analysis was conducted for unraveling the putative targets involved in the observed effects. The flowchart (Figure 1) of the study is reported below and schematizes the different phases of the present study, which supports the use of the formulation as an innovative tool to face the burden of inflammation and to combat the osteoclastogenic process occurring in postmenopausal women.

## 2. Materials and Methods

Dry water extracts from aerial parts of *Equisetum arvense* L. (10% silica), seeds of *Glycine max* L. Merr. (25% genistein, 40% total isoflavones), flowers and leaves of *Crataegus curvisepala* Lind. (2.5% total flavanoids), fruits of *Vitex agnus-castus* L. (0.5% agnuside), resveratrol from roots of *Polygonum cuspidatum* Siebold & Zucc., vitamin K2 (menaquinone), vitamin D3 (cholecalciferol), and magnesium oxide powder were provided by Cristalfarma S.r.l. (Milan, Italy).

The dry extracts and powders were rehydrated in water via ultrasound-assisted extraction (Trans-sonic T460 ultrasonic bath; Elma, Singen, Germany). The formulation concentration after rehydration was 10 mg/mL. Components were used in combination, with the proportions reflecting the composition of the commercial food supplement Zelda GOLD^®^ property of Cristalfarma S.r.l. (Milan, Italy), at the following percentages: *Equisetum arvense* 35.32%, magnesium 23.18%, *Glycine max* 17.66%, *Crataegus curvisepala* 17.66%, *Vitex agnus-castus* 3.53%, resveratrol 2.65%, vitamin K 0.009%, and vitamin D3 0.004%.

### 2.1. HPLC

The formulation was subjected to a reverse phase HPLC-UV analysis, in gradient elution mode, to quantitatively determine the phenolic composition. The HPLC apparatus consisted of a two PU-2080 PLUS chromatographic pump, a DG-2080-54-line degasser, a mix-2080-32 mixer, UV, an AS-2057 PLUS autosampler, and a CO-2060 PLUS column thermostat (all from Jasco, Tokyo, Japan). ChromNAV2 Chromatography software (version 2.2.2.3) was used for integration.

The separation was conducted within 60 min of the chromatographic run, starting from the following separation conditions: 97% water with 0.1% formic acid and 3% methanol with 0.1% formic acid. The details about gradient are listed in Table S1 (Supplementary Materials). The separation was performed on an Infinity lab Poroshell 120-SB reverse phase column (C18, 150 × 4.6 mm i.d., 2.7 µm; Agilent, Santa Clara, CA, USA). The column temperature was set at 30 °C. The quantitative determination of the phenolic compounds was performed via a UV detector at 254 nm. The injection volume was 5 µL. Quantification was carried out using 7-point calibration curves, with linearity coefficients (R^2^) > 0.999, in the concentration range of 2–140 µg/mL. The final concentration of the extract was 40 mg/mL before injecting it into the HPLC system.

### 2.2. Colorimetric Assays

The total phenolic and flavonoid contents were determined using the Folin–Ciocalteu method, with the results reported as equivalents of gallic acid (mg GAE/g dry extract) and rutin (mg RE/g dry extract). The antioxidant activity of the formulation was evaluated through the 2,2-azinobis (3-ethylbenzothiazoline-6-sulfonic acid) ABTS assay. The comprehensive procedures are reported in the literature [29] and in the Supplementary Materials.

### 2.3. Ecotoxicological Investigation

#### 2.3.1. Preparation of Test Sample Concentrations

A stock solution of the formulation was prepared at a concentration of 10 mg/mL, and subsequently diluted to achieve the required concentration range for testing. Distilled water was used for dilutions in the allelopathy assay, whereas artificial seawater was utilized for the brine shrimp lethality assay, maintaining a concentration range of 0.625 to 10 mg/mL in both assays.

#### 2.3.2. Allelopathy Assay

An experiment using Petri dishes was designed to investigate the phytotoxic effects of the tested formulation within a concentration range of 0.62 to 10 mg/mL. The seeds of *Cichorium intybus* (CI), *Dichondra repens* (DR), and *Raphanus sativus* (RS), which are commercially available dicotyledons, were chosen, due to their rapid germination and high sensitivity to external agents. These traits make them reliable indicators for detecting subtle inhibitory effects. By incorporating these species, the study aimed to comprehensively evaluate the formulation’s potential to impact different plant species and determine whether it exerts broad-spectrum or selective phytotoxicity.

The assay was conducted in 90 mm Petri dishes lined with double-layered filter paper disks, which were soaked with 3 mL of the extract at varying concentrations. Distilled water was used as the negative control. The seeds were sterilized by soaking them in a diluted bleach solution (NaClO:dH_2_O, 1:9) for 10 min, followed by thorough rinsing with sterile distilled water to remove residual bleach. Ten seeds from each plant type were placed on the filter paper disks, with the Petri dishes divided into three sections to separate the seed varieties. The dishes were sealed with parafilm to maintain a controlled environment and incubated in darkness at a temperature of 25 ± 2 °C for 96 h.

Germination was identified through the appearance of a radicle extending at least 2 mm and showing a natural geotropic curvature, while seeds that only swelled without full germination were excluded. The length of the seedlings was measured and classified into three categories: low (<0.4 cm), medium (0.5–0.9 cm), or high (>1 cm). After 96 h, the germination rate and seedling lengths were recorded. Results were reported as Germination Percentage (GP) and Seedling Length (SL), calculated as the average seedling length relative to the control group.

#### 2.3.3. Brine Shrimp Toxicity Bioassay

Cysts of *Artemia salina* L. were incubated in oxygenated artificial seawater (1 g cysts/L) with a salinity of 32 g/L to facilitate hatching. After 24 h, the toxicity evaluation was performed using the brine shrimp lethality assay (BSLA), as outlined by Meyer et al. [30] and McLaughlin et al. [31], employing freshly hatched nauplii. Triplicate tests were conducted for each concentration, ranging from 0.625 to 10 mg/mL. Each glass tube contained 5 mL of artificial seawater and 10 nauplii. After 24 h of exposure, the number of surviving nauplii was recorded, and the mortality rate was calculated using the following formula: ((T − S)/T) × 100, where T represents the total number of larvae introduced, and S denotes the number of surviving nauplii. The median lethal concentration (LC50) was determined using GraphPad Prism™ (Version 5.01) software (GraphPad Software, Inc., San Diego, CA, USA). The test was considered valid if mortality in the control group did not exceed 10%.

#### 2.3.4. *Daphnia magna* Cardiotoxicity Assay

For each experimental group, three non-pregnant *Daphnia magna* specimens were selected and allocated to individual wells of a six-well plate, with three specimens per well. One well contained spring water as the control group, while another contained the formulation at a concentration of 6.866 mg/mL, equivalent to the LC_50_ value determined in the previous BSLA experiment. The specimens were exposed to the assigned treatments for 15 min at room temperature. After the exposure period, each *D. magna* specimen was individually transferred to a microscope slide containing a 50 μL droplet of the tested formulation, and their heartbeat rates were observed and recorded under a microscope for 15 s. To assess the cardiotoxic response, the specimens were subsequently exposed to a 10% ethanol solution for 2 min.

All observations were conducted in triplicate, and the experiment was repeated three times for consistency. The results, specifically the decrease in heart rate, were compared across the untreated group (negative control), the ethanol-treated group (positive control), and the group treated with the tested formulation.

### 2.4. In Vitro Study

#### 2.4.1. Cell Culture

C2C12 myoblasts, a murine skeletal muscle cell line commonly used as a model for oxidative stress and inflammatory responses, were chosen for this study due to their high sensitivity to pro-oxidant stimuli, such as hydrogen peroxide (H_2_O_2_). The cells were cultured in DMEM (Dulbecco’s Modified Eagle Medium) supplemented with 20% fetal bovine serum (FBS) and 1% penicillin−streptomycin, in a humidified atmosphere of 5% CO_2_ at 37 °C. Cells were routinely monitored under an inverted microscope to ensure proper morphology and confluence. Upon reaching approximately 70% confluence, cells were detached using 0.05% trypsin-EDTA, passaged, and seeded at appropriate densities for subsequent assays.

#### 2.4.2. Determination of Cell Viability

The MTT assay was used to evaluate the effect of the tested formulation on the viability of C2C12 cells. This assay, widely employed to assess cell viability and cytotoxicity, relies on the reduction of MTT (3-(4,5-dimethylthiazol-2-yl)-2,5-diphenyltetrazolium bromide) by mitochondrial dehydrogenases in viable cells to produce insoluble formazan crystals. The purple formazan was dissolved in DMSO and quantified spectrophotometrically at 570 nm.

To assess cell viability under baseline conditions, C2C12 cells were seeded in 96-well plates at a density of 5 × 10^3^ cells/well and allowed to adhere for 24 h. The formulation was then administered at final concentrations of 200, 500, and 1000 µg/mL for 24 h. After treatment, MTT solution (5 mg/mL) was added to each well and incubated for 3 h at 37 °C. Formazan crystals were solubilized in DMSO, and absorbance was measured at 570 nm using a microplate reader. Control wells containing medium without cells were used as blanks, and cell viability was calculated relative to the untreated control wells.

For evaluating the effect of the formulation under H_2_O_2_-induced oxidative stress, the same experimental setup was used. Following 24 h treatment with the formulation, H_2_O_2_ was added at final concentrations of 500 µM for 3 h to induce oxidative stress. After H_2_O_2_ exposure, the medium was replaced with MTT solution (5 mg/mL) and incubated for 3 h at 37 °C. Formazan crystals were dissolved in DMSO and absorbance was measured at 570 nm. The control wells containing medium without cells were used as blanks, and cell viability was determined relative to the untreated control wells.

### 2.5. Gene Expression Analysis

Total RNA was extracted from C2C12 cells using TRI reagent (Sigma-Aldrich, St. Louis, MO, USA), according to the manufacturer’s protocol, and reverse transcribed using a High-Capacity cDNA Reverse Transcription Kit (Thermo Fisher Scientific, Waltman, MA, USA). Specifically, 1 μg of total RNA extracted from each sample in a 20 μL reaction volume was reverse transcribed using a High-Capacity cDNA Reverse Transcription Kit (Applied Biosystems, Foster City, CA, USA). Reactions were incubated in a 2720 Thermal Cycler (Applied Biosystems, Foster City, CA, USA) initially at 25 °C for 10 min, then at 37 °C for 120 min, and finally at 85 °C for 5 s. Gene expression was determined by quantitative real-time PCR using TaqMan probe-based chemistry. PCR primers and TaqMan probes were purchased from Thermo Fisher Scientific Inc. The Assays-on-Demand Gene Expression Products used for gene expression evaluations in the mouse cortex specimens were as follows: Mm00446190_m1 for IL-6 gene, Mm04336676_m1 for PRLR, Mm00433149_m1 for ESR1, Mm00443258_m1 for TNFα, Mm00441908_m1 for RANKL, and Mm0607939_s1 for β-actin gene. β-actin was used as the housekeeping gene. The elaboration of the data was conducted using the Sequence Detection System (SDS) software version 2.3 (Thermo Fisher Scientific). The relative quantification of gene expression was performed using the comparative 2^−∆∆Ct^ method.

### 2.6. In Silico Study

Direct (physical) and indirect (functional) protein–protein interactions were predicted using the bioinformatics platform STRING [32], and the results have been reported as the protein–protein network. The prediction was limited to the species Homo sapiens, and the interaction score was set to confidence 0.4 as the threshold.

### 2.7. Statistical Analysis

The data represent the group means ± S.D. of three to five experiments performed in triplicate. Statistical analysis was performed using GraphPad Prism™ (Version 5.01) software (GraphPad Software, Inc., San Diego, CA, USA). The statistical significance (*p* < 0.05) was evaluated through analysis of variance (ANOVA) followed by Newman–Keuls comparison multiple tests.

## 3. Results

### 3.1. Phytochemical Analysis

**Figure 2:**
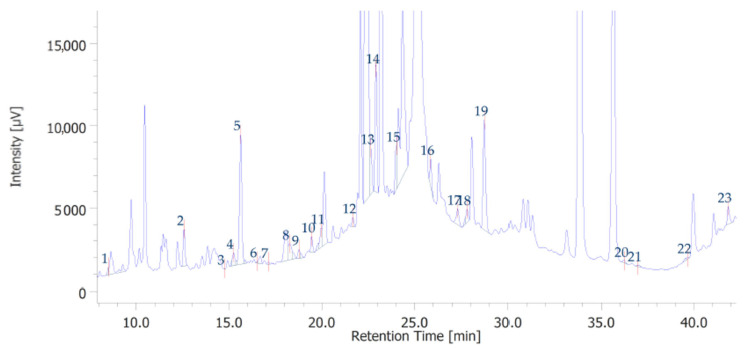
Chromatographic analysis of the phenolic composition of the formulation. The separation of the phenols was conducted in gradient elution mode on an Infinity lab Poroshell 120-EC reverse phase column (C18, 150 mm × 4.6 mm i.d., 2.7 µm; Agilent Santa Clara, CA, USA). Details about the chromatographic conditions are reported in Table S1.

**Figure 3:**
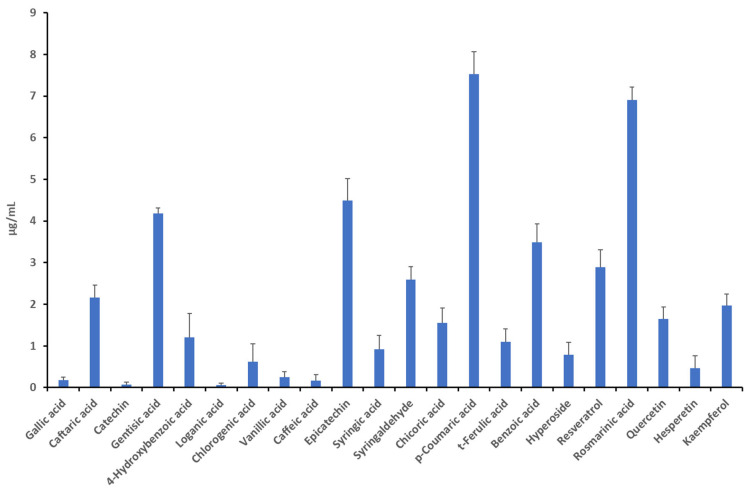
Quantitative determination chromatographic analysis of the phenolic composition of the formulation. Among the identified phenolic compounds, the prominent ones were caftaric acid (peak #2, Rt 12.575 min.), gentisic acid (peak #4, Rt 15.242), epicatechin (peak #10, Rt 19.442), syringakdehyde (peak #12, Rt 21.658), p-coumaric acid (peak #14, Rt 22.917), benzoic acid (peak #16, Rt 25.850), resveratrol (peak #18, Rt 27.817), rosmarinic acid (peak #19, Rt 28.742), quercetin (peak #20, Rt 36.285), and kaempferol (peak #23, Rt 41.817).

The formulation was analyzed to determine the content of phenolic compounds. The colorimetric determination of the total phenols and flavonoids, expressed as gallic acid and rutin, respectively, showed that phenolic acids were four times more concentrated than flavonoids in the formulation (Table 1). This is also consistent with the chromatographic analysis (Figure 2 and Figure 3) that demonstrated phenolic acids, such as gentisic acid (peak #4, Rt 15.242) and p-coumaric acid (peak #14, Rt 22.917), and hydrocinnamic acids, such as caftaric acid (peak #2, Rt 12.575 min.) and rosmarinic acid (peak #19, Rt 28.742), as the main phytochemicals present in the formulation. The scavenging reducing properties of the formulation were tested through the ABTS assays, which showed antioxidant activity, measured as the IC_50_ value of 0.42 mg/mL (Table 2).

### 3.2. Allelopathy Assay

**Figure 4:**
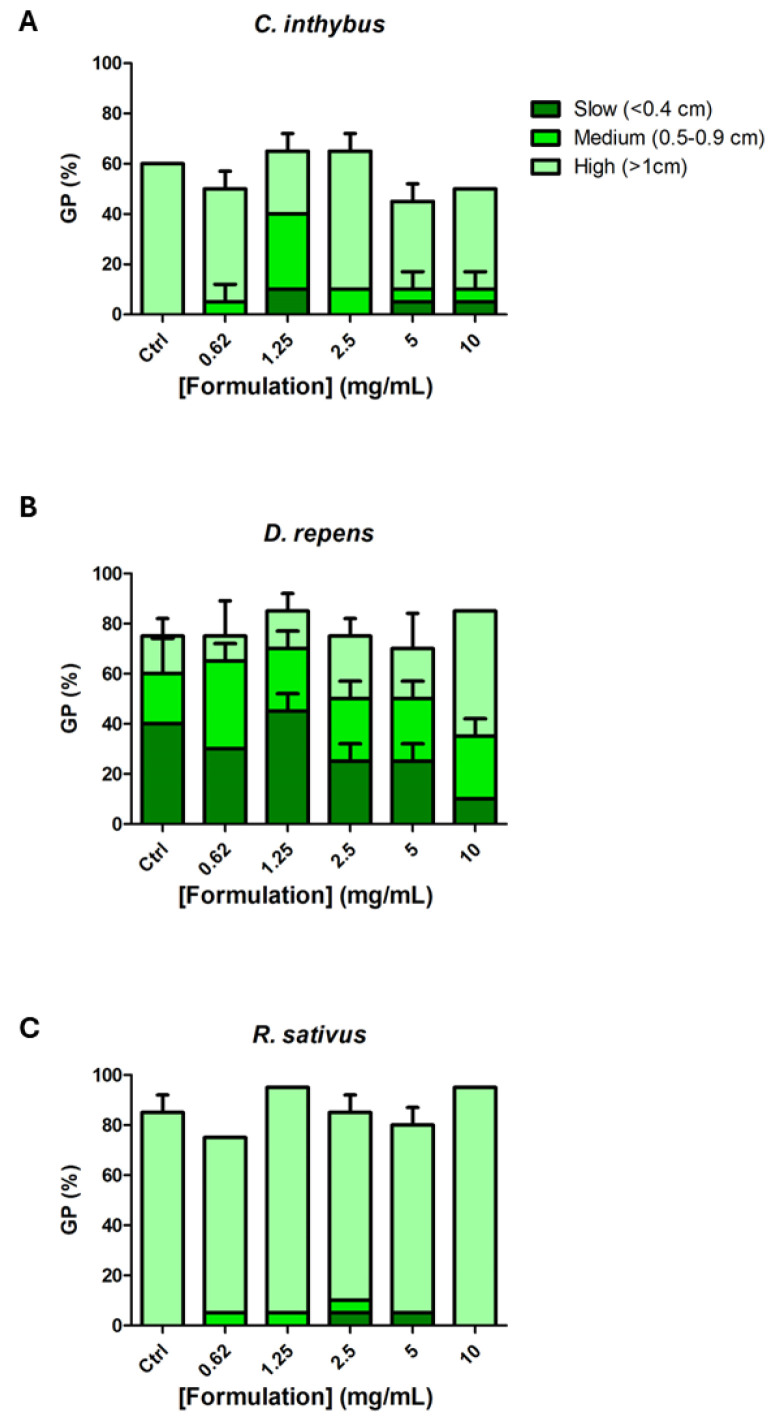
Effect of the tested formulation (0.62 to 10 mg/mL) on germination percentage (GP%) and seedling elongation of *Cichorium intybus* (**A**), *Dichondra repens* (**B**), and *Raphanus sativus* (**C**). Seedling lengths are categorized into three growth groups: slow (<0.4 cm), medium (0.5–0.9 cm), and high (>1 cm).

The biocompatibility of the formulation was measured via allelopathy assay. In particular, seeds of the dicotyledon species *C. inthybus*, *D. repens*, and *R. sativus* were challenged with scalar concentrations of the formulation (0.62–10 mg/mL). The formulation did not exert any relevant alteration of germination percentage (Figure 4), whilst a slight reduction in root elongation was observed in the *C. inthybus* species (Table 3).

### 3.3. Brine Shrimp Lethality Assay

**Figure 5:**
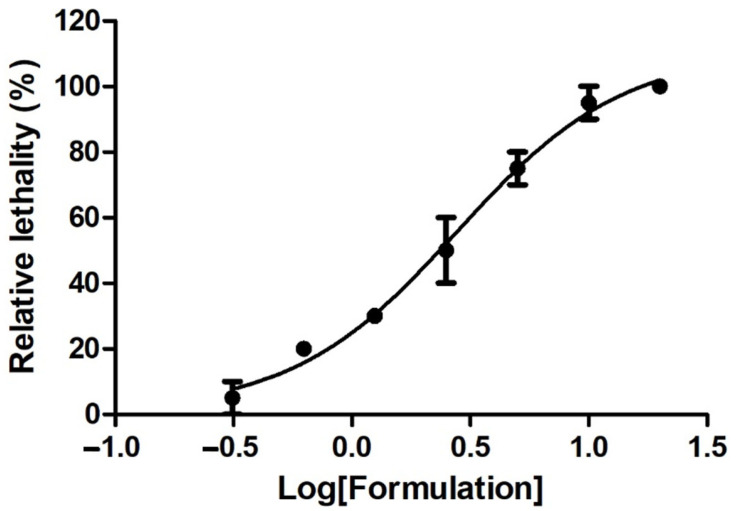
Dose–response curve displaying the lethality effects induced by the tested formulation (0.625–10 mg/mL) on brine shrimps (*Artemia salina*). LC_50_ value was 2.783 mg/mL.

In the brine shrimp (*A. salina*) lethality assay, the crustaceans were exposed to scalar concentrations of the formulation (0.625–10 mg/mL), and toxicity was measured in terms of the LC_50_ value. The results reported in Table 4 and Figure 5 indicate the biocompatibility of the formulation that presented an LC_50_ of 2.873 mg/mL, beyond the limit of biocompatibility (1000 µg/mL) of Meyer and Clarkson’s toxicity scales.

### 3.4. Daphnia Magna Cardiotxocity Assay

**Figure 6:**
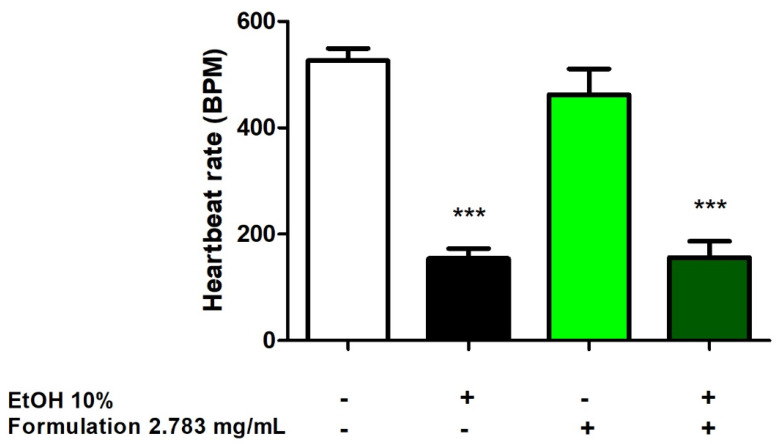
Acute exposure of the Daphnia magna to the formulation at 2.783 mg/mL showed no cardiotoxic effect, and no cardioprotective effect was observed when co-treated with 10% ethanol as a cardiotoxic stimulus. Data are reported as means ± SEM. ANOVA, *p* < 0.0001. *** *p* < 0.001 vs. negative control group.

In the *Daphnia magna* cardiotoxicity assay, the crustaceans were challenged with the formulation (2.783 mg/mL) in the presence and absence of ethanol 10% as a cardiotoxic stimulus, and heart rate reduction was evaluated. The results indicated the null effect of the formulation on *D. magna* heart rate (Figure 6).

### 3.5. Protective Effects on C2C12 Cells

**Figure 7:**
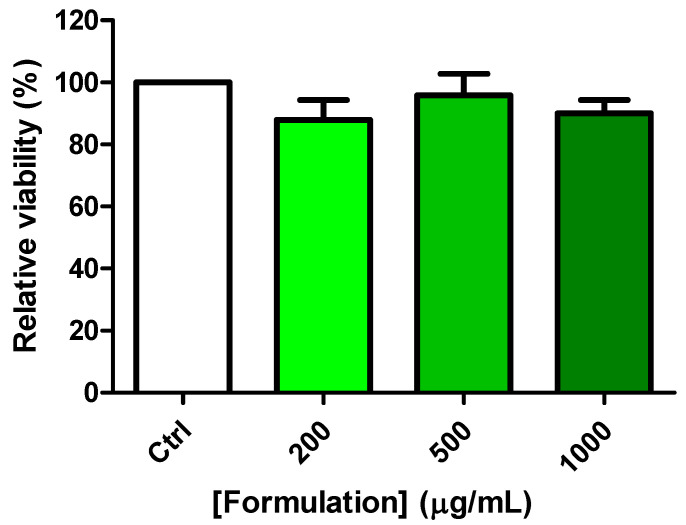
Effect of the formulation (200–500 µg/mL) on C2C12 cell viability.

**Figure 8:**
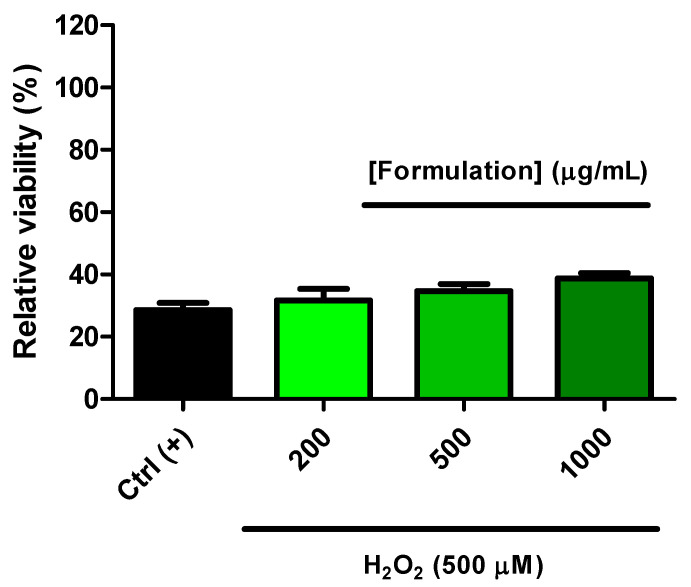
Effect of the formulation (200–500 µg/mL) on the viability of C2C12 cells exposed to hydrogen peroxide (H_2_O_2_).

**Figure 9:**
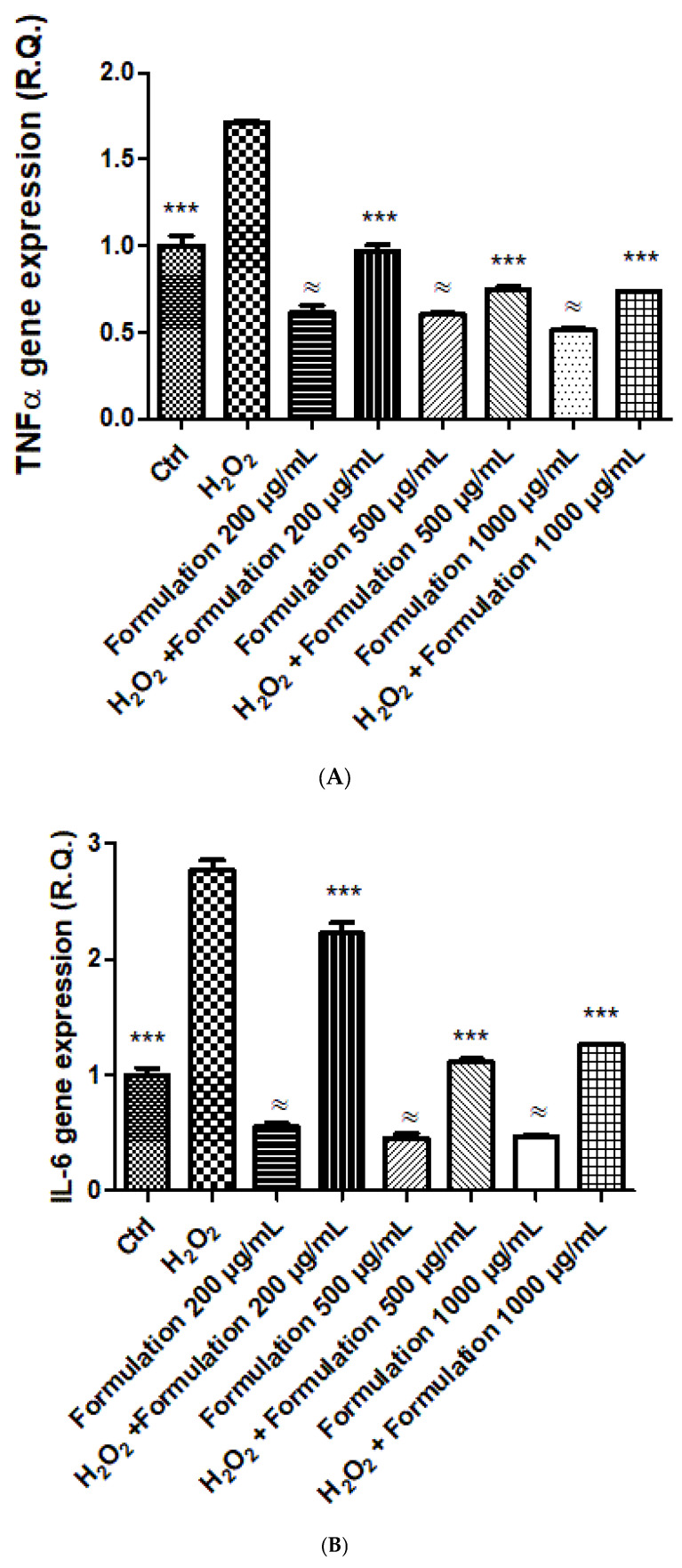

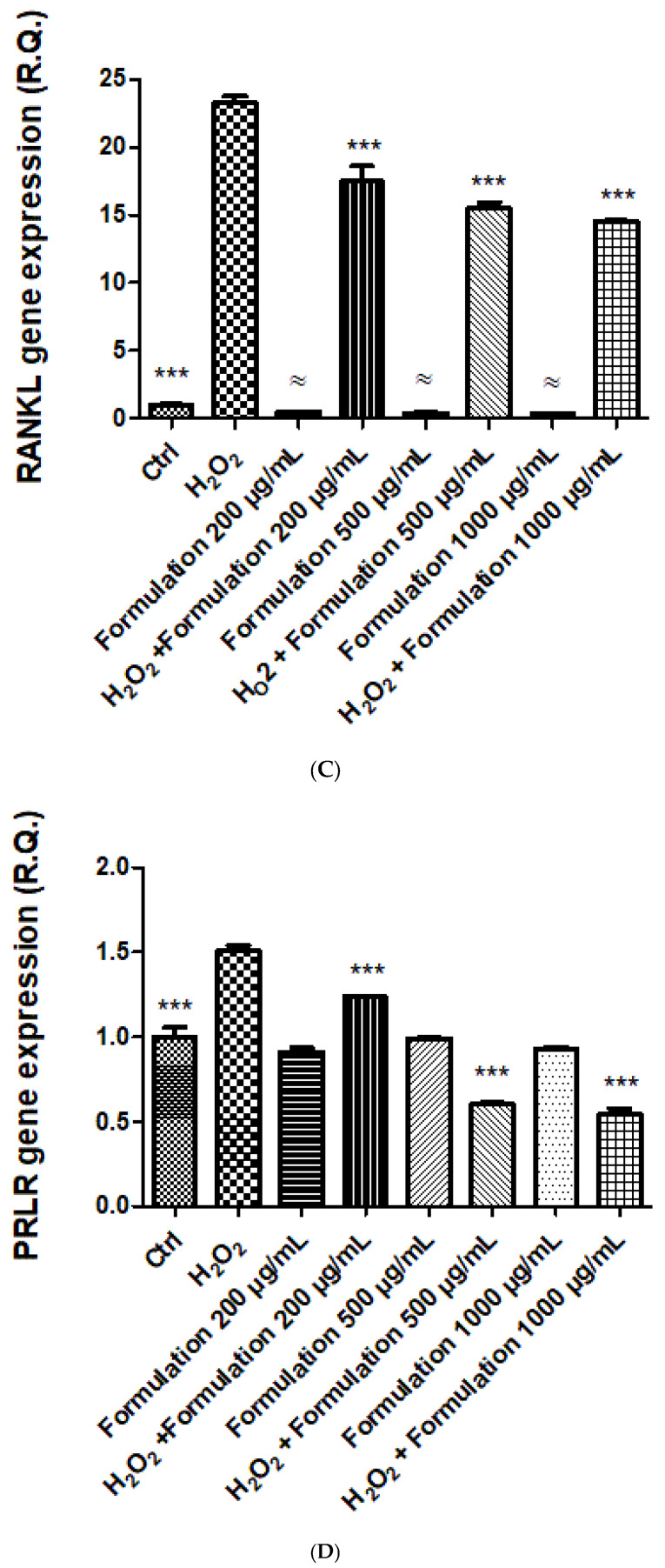

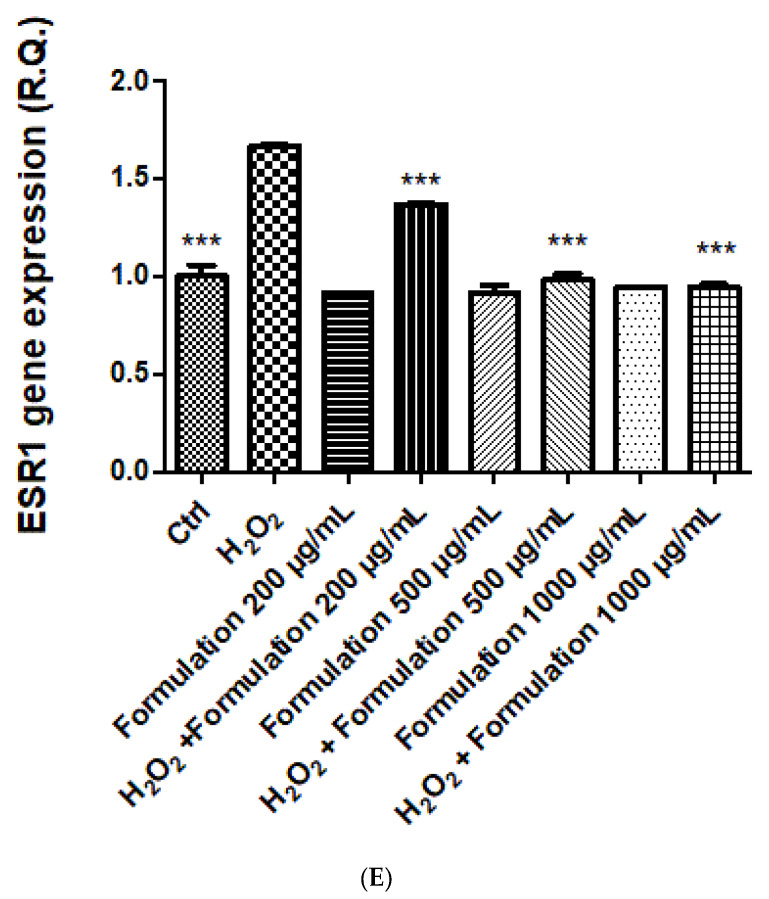
(**A**) Effect of the formulation (200–500 µg/mL) on the gene expression of TNFα in C2C12 cells exposed to hydrogen peroxide (H_2_O_2_). ANOVA, *p* < 0.0001; ≈ *p* < 0.001 vs. Ctrl, *** *p* < 0.001 vs. hydrogen peroxide. (**B**) Effect of the formulation (200–500 µg/mL) on the gene expression of IL-6 in C2C12 cells exposed to hydrogen peroxide (H_2_O_2_). ANOVA, *p* < 0.0001; ≈ *p* < 0.001 vs. Ctrl, *** *p* < 0.001 vs. hydrogen peroxide. (**C**) Effect of the formulation (200–500 µg/mL) on the gene expression of RANKL in C2C12 cells exposed to hydrogen peroxide (H_2_O_2_). ANOVA, *p* < 0.0001; ≈ *p* < 0.001 vs. Ctrl, *** *p* < 0.001 vs. hydrogen peroxide. (**D**) Effect of the formulation (200–500 µg/mL) on the gene expression of PRLR in C2C12 cells exposed to hydrogen peroxide (H_2_O_2_). ANOVA, *p* < 0.0001; *** *p* < 0.001 vs. hydrogen peroxide. (**E**) Effect of the formulation (200–500 µg/mL) on the gene expression of ESR1 in C2C12 cells exposed to hydrogen peroxide (H_2_O_2_). ANOVA, *p* < 0.0001; *** *p* < 0.001 vs. hydrogen peroxide.

The cells were treated with the formulation (200–1000 µg/mL) both under basal conditions and after exposing the cells to the oxidative stress stimulus consisting of hydrogen peroxide (500 µM). The formulation did not influence cell viability in either basal or oxidative stress conditions (hydrogen peroxide) (Figure 7 and Figure 8). In cells exposed to hydrogen peroxide, the formulation was effective in blunting the upregulated gene expression of TNFα, IL-6, RANKL, ESR1, and PRLR (Figure 9A–E). In basal conditions, the formulation was effective in reducing the gene expression of TNFα (Figure 9A), IL-6 (Figure 9B), RANKL (Figure 9C), and PRLR (Figure 9D), without any relevant change to ESR1 (Figure 9E).

### 3.6. Bioinformatics

**Figure 10:**
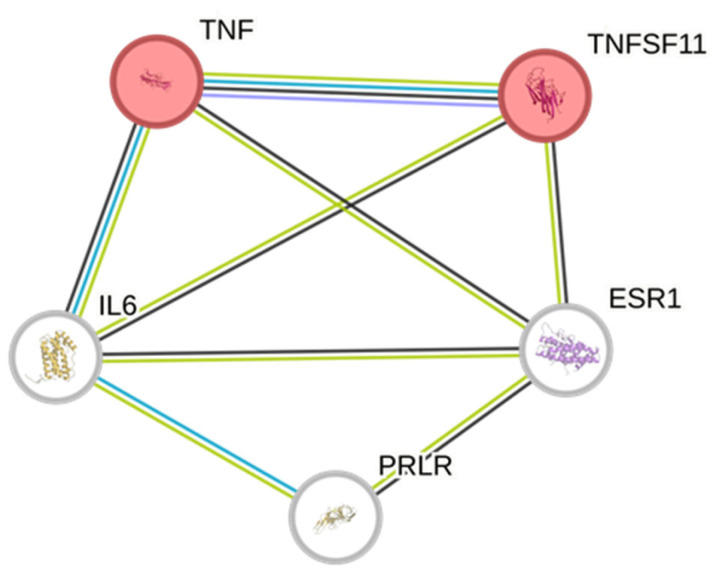
Protein–protein interactions predicted by the platform STRING. The colored nodes representing TNFα and RANKL (also known as TNFSF11) could represent first shells for interactors.

A bioinformatics prediction was conducted on the platform STRINGH for unraveling putative interactions between TNFα, IL-6, RANKL, ESR1, and PRLR. In Figure 10, the colored nodes representing TNFα and RANKL (also known as TNFSF11) could represent first shells for interactors.

## 4. Discussion

In the present study, a novel commercial formulation based on water extracts from *E. arvense* (10% silica), *V. agnus-castus*, *C. curvisepala*, and *G. max*, standardized in agnuside, genistein, total flavonoids, and rosmarinic acid, respectively, was evaluated. The formulation also included resveratrol from the roots of *P. cuspidatum*, vitamin K2, and vitamin D3. The ingredients were selected for managing postmenopausal disorders, including osteoporosis and vasomotor symptoms [13,14,15,16,17]. A phytochemical analysis was conducted for determining the formulation’s content of phenolic compounds and flavonoids, which have long been known to possess antioxidant and anti-inflammatory properties [33]. Hydroxycinnamic and benzoic acids have been reported to be ubiquitous in angiosperms [34]. However, it is important to highlight that in at least two medicinal plants present in the formulation, namely *E. arvense* and *V. agnus-castus*, together representing almost 40% of the formulation weight, hydroxycinnamic and benzoic acids are among the main phenolic compounds [35,36]. This could explain, at least in part, the phenolic profiles shown in Figure 2 and Figure 3 [37]. The content of total phenols is also consistent with the antioxidant effect reported in Table 2 [38]. In fact, the formulation displayed antioxidant effects in the ABTS assay, one of the most reliable methods for evaluating the radical scavenger properties of antioxidants, which represents the main mechanism of lipid oxidation inhibition. This is related, albeit partially, to the very fast single electron transfer reactions between phenolic compounds and the ABTS assay [39]. Additionally, the antiradical effects exerted by the formulation could also account for the anti-inflammatory effects [33,40,41,42].

The present research also included the biocompatibility limit evaluation through the use of validated and independent ecotoxicological experimental paradigms, namely the allelopathy, *Artemia salina* lethality, and *Daphnia magna* cardiotoxicity assays. These models have a consolidated use in toxicology and pharmacology for predicting toxicity and defining dose range in eukaryotic organisms [37,43,44].

The allelopathy assay, while traditionally utilized to study plant–plant interactions, has been employed in this study as a preliminary tool for evaluating the biocompatibility of natural formulations. Its integration into a multi-step assessment framework allows for the early identification of potential toxicological effects by measuring germination percentage and seedling growth, two highly sensitive biological endpoints. These parameters can reveal subtle inhibitory or cytotoxic effects that might not be evident in more complex systems. A formulation demonstrating minimal effects on germination and seedling elongation would provide an initial indication of its biocompatibility, supporting its progression to further stages of evaluation, such as cellular or animal models [45]. The allelopathy test also serves as a cost-effective and resource-efficient method, offering rapid insights while minimizing the need for more labor-intensive and ethically sensitive studies. In the present study, the formulation under investigation showed minimal effects on germination percentages (Figure 4) for the three tested species, across all tested concentrations (0.62–10 mg/mL), indicating the absence of significant phytotoxicity at this early developmental stage. This suggests that the formulation does not interfere with the initial metabolic processes required for seed germination. Root elongation responses (Table 3) varied across species, with *C. intybus* exhibiting a slight inhibitory effect at higher concentrations, while *D. repens* and *R. sativus* demonstrated tolerance or even slight stimulation. These differences may reflect species-specific sensitivities and highlight the potential influence of secondary metabolites, such as phenolic and flavonoid compounds, present in the formulation.

In the brine shrimp (*A. salina*) lethality assay, the toxicity level of the formulation was determined as the LC_50_ (lethality concentration) [45], applying the classifications of Meyer and Clarkson. Specifically, the formulation was considered non-toxic and toxic based on the Meyer classification for LC_50_ values higher and lower than 1000 µg/mL, respectively [31], whilst based on the the Clarkson classification [46], four levels of toxicity have been suggested, as listed below:

− Non-toxic for LC_50_ > 1000 µg/mL;
− Low toxicity for LC_50_ between 500 µg/mL and 1000 µg/mL;
− Toxic for LC_50_ between 100 µg/mL and 500 µg/mL;
− Highly toxic for LC_50_ < 100 µg/mL.

According to these criteria, the formulation can be considered non-toxic, with LC_50_ values of 2.783 mg/mL (Figure 5; Table 4).

The formulation was also tested using the *D. magna* assay at a concentration of 2.783 mg/mL, corresponding to the LC_50_ calculated via the brine shrimp test. Also, in this experimental paradigm, the formulation was biocompatible, without any alteration of the heart rate in basal conditions. However, it was not effective at reverting the ethanol (10%)-induced reduction in heart rate, thus excluding any cardioprotective effect (Figure 6).

On the basis of the results yielded by *A. salina* and *D. magna* toxicity assays, a concentration range almost 3-fold lower (1000 µg/mL) was employed for investigating the protective effects of the formulation on C2C12 cells. The formulation was well tolerated by the cells in the selected concentration range, without any significant alteration in cell viability (Figure 7). In the same range, the protective effect of the formulation was evaluated against hydrogen peroxide. Although it was not statistically significant, a tendency towards counteracting the cytotoxicity induced by hydrogen peroxide was observed, especially at the highest tested concentration (Figure 8). This is consistent with the intrinsic radical scavenger properties of the formulation observed in the ABTS assay and related, albeit partially, to the total phenols and flavonoids [39]. In C2C12 cells, the gene expression of the cytokines TNFα and IL-6 was assessed as well. The evaluation of cytokine levels is of particular relevance, due to their increased levels during menopause; indeed, cytokines increase osteoclast function and bone resorption [26], thus indicating the reduction of the inflammatory burden as a pivotal strategy for postmenopausal bone health. The gene expression of these biomarkers was increased in the presence of the hydrogen peroxide stimulus (Figure 9A–E), and this is consistent, albeit partially, with the literature [47,48]. The formulation was able to reduce the gene expression, especially in conditions of oxidative stress, thus suggesting its potential use as an inhibitor of bone resorption. The inhibition of the gene expression of IL-6 and TNFα agreed with the total content of phenols and flavonoids in the formulation [47]. Indeed, phenolic compounds are capable of reducing the gene expression of cytokines, including IL-6 and TNFα, in vitro, in macrophages exposed to an inflammatory stimulus [49]. During menopause, there is also an increase in the levels of RANKL that stimulates and inhibits osteoclasts and osteoblasts, respectively [27], thus leading to bone resorption and osteoporosis. The gene expression of RANKL was also assessed, and the inhibition of its gene expression could be related, at least in part, to the pool of hydroxycinnamic acids that have been suggested to play a master role in inducing anti-osteoporotic effects through the inhibition of the RANKL pathway [50]. In the same experimental conditions, the gene expression of ESR1 and PRLR, exerting stimulating and inhibiting effects on bone formation, respectively [24,25], was also assessed. Regarding ESR1, the formulation did not alter the basal gene expression of the receptor, whilst it restored its basal gene expression in the presence of oxidative stress (Figure 9E). The reduction in ESR1 gene expression is consistent, at least in part, with the content of resveratrol in the formulation [51]. Also, in the case of PRLR, the formulation did not modify the basal receptor gene expression. However, in cells exposed to hydrogen peroxide, a significant reduction in PRLR gene expression, even under the basal threshold (Ctrl), was observed, thus further corroborating the potential inhibitory role of the formulation in the osteoclastogenic process. In this context, pivotal roles could also be played by the contents of vitamin K2 and D3 in the formulation [52].

Finally, a bioinformatics prediction was conducted on the platform STRINGH that showed direct interactions among all the proteins whose gene expression was evaluated in C2C12 cells (Figure 10). In this context, the colored nodes representing TNFα and RANKL (also known as TNFSF11) could represent first shells for interactors, thus playing a prominent position in the scenario of the putative interactions between formulation compounds and human targets, with possible influences on numerous pathways, including osteoclast differentiation (hsa04380; Figure S1, Supplementary Materials).

The efficacy of the present formulation in reducing the gene expression of the biomarkers playing master roles in inflammation and osteoclast function activation in postmenopausal women agrees with previous in vivo findings of ours pointing to the capability of the association of *E. arvensis*, lactoferrin, soy isoflavones, and vitamin D3 in improving bone homeostasis in both young and aged rats [18], thus further corroborating the rationale for the pharmacological association of medicinal plants and natural compounds with counteracting age-related conditions, including postmenopausal disorders, that are characterized by multifactorial metabolic alterations [12].

## 5. Conclusions

In the present study, the protective effects of an innovative formulation consisting of vitamin K2, vitamin D3, resveratrol from roots of *P. cuspidatum*, and water extracts from *E. arvense*, *C. curvisepala*, *V. agnus-castus*, and *G. max* were evaluated on C2C12 cells exposed to hydrogen peroxide. The formulation blunted the hydrogen peroxide-induced upregulation of different factors involved in bone homeostasis, namely TNFα, IL-6, RANKL, ESR1, and PRLR. The content of vitamins and total phenolic compounds, particularly hydroxycinnamic acids and resveratrol, could explain, at least in part, the effectiveness of the formulation that also, according to the bioinformatics prediction conducted using the platform STRINGH, could influence osteoclast differentiation through an inhibitory effect. Overall, the results of the study corroborate the use of the formulation for counteracting the oxidative stress and inflammation at the basis of the osteoclastogenic process, thus potentially restoring bone homeostasis. Despite this efficacy, the use of an in vitro model, namely C2C12 cell lines, has the limitation of a lack of inter-tissue communication present in the entire organism. Therefore, future directions of research will target the evaluation of the efficacy of in vivo and clinical paradigms. Of particular relevance is the role of aging, which future studies should consider, as it is closely related to menopausal disorders in the response to the formulation and in different treatment groups characterized by different ages, namely pre- and postmenopausal groups.

## Figures and Tables

**Table 1:** Total phenolic content and flavonoid content of the tested formulation. Values are reported as mean ± S.D. of three parallel measurements. GAE: gallic acid equivalents; RE: rutin equivalents; and de: dry extract.

Parameters	Results
Total phenolic content (mg GAE/gde)	29.30 ± 1.30
Total flavonoid content (mg RE/gde)	6.43 ± 0.56

**Table 2:** Antioxidant capacity of the tested formulation. Values are reported as mean ± S.D. of three parallel measurements.

Sample	ABTS
Formulation	0.42 ± 0.06
Trolox	0.03 ± 0.01

**Table 3:** Seedling length responses of *Cichorium inthybus*, *Dichondra repens*, and *Raphanus sativus* seeds under varying formulation concentrations (range: 0.62–10 mg/mL), expressed in centimeters. Data are presented as means ± SD.

Seedling Length (cm)			
Treatments (mg/mL)	*C. inthybus*	*D. repens*	*R. sativus*
0 (Ctrl)	28.83 ± 3.77	5.79 ± 0.11	35.89 ± 1.40
0.62	29.96 ± 4.54	8.69 ± 3.10	33.56 ± 3.62
1.25	19.38 ± 8.55	5.79 ± 0.77	35.27 ± 5.90
2.5	27.63 ± 6.78	8.00 ± 0.00	28.65 ± 1.44
5	19.90 ± 1.27	7.64 ± 2.73	29.94 ± 7.16
10	17.60 ± 3.96	14.81 ± 0.43	36.02 ± 0.45

**Table 4:** Brine shrimp lethality assay results in terms of LC_50_ value and toxicity levels according to Meyer’s and Clarkson’s classifications. Tested samples: formulation in a concentration range between 0.625 and 10 mg/mL.

Tested Extract	Concentration Range (mg/mL)	LC_50_	95% Confidence Interval	R^2^	Toxicity Class	
					Meyer’s classification	Clarkson’s classification
Formulation	0.625–10	2.783	1.812–4.273	0.975	Non-toxic	Non-toxic

## Data Availability

The original contributions presented in this study are included in the article/Supplementary Materials. Further inquiries can be directed to the corresponding author.

## Supplementary Materials

The following supporting information can be downloaded at: https://www.mdpi.com/article/10.3390/foods14050896/s1. Materials and methods of colorimetric assays. Table S1: Gradient elution condition for chromatographic determination (HPLC-UV) analysis of phenolic compounds. UV detector was set at 254 nm; Figure S1: Osteoclast differentiation pathway.
