# Supplementary material for: A Novel Formulation Based on Resveratrol and Water Extracts from Equisetum arvense, Crataegus curvisepala, Vitex agnus-castus, and Glycine max Inhibits the Gene Expression of Inflammatory and Osteoclastogenic Biomarkers on C2C12 Cells Exposed to Oxidative Stress
Source: Foods. 2025 Mar 6;14(5):896. doi: 10.3390/foods14050896 (PMC11899544; doi:10.3390/foods14050896)
Supplement: Supplementary file 1 [file foods-14-00896-s001.zip › foods-3481095-supplementary.pdf]

Colorimetric assays

ABTS Assay: The antioxidant potential of the extracts and Eos was evaluated using the ABTS (2,2′-azino-bis(3-ethylbenzothiazoline)-6-sulfonic acid) radical scavenging assay. The reaction of 7 mM ABTS solution with 2.45 mM potassium persulfate resulted in the generation of the $ABTS^+$ radical cation. This reaction mixture was left to stand in the dark at RT for 12–16 hours to ensure the formation of the stable $ABTS^+$ radical. Before proceeding with the assay, the $ABTS^+$ solution was diluted with methanol and adjusted to an absorbance value of 0.700 at 734 nm, ensuring consistent results. One mL of the sample solution was added to 2 mL of the prepared $ABTS^+$ solution followed by gentle mixing. The mixture was incubated at RT for 30 minutes to ensure the reaction between the sample and the $ABTS^+$ radicals. Then the spectrophotometer was used to measure the absorbance of the solution at 734 nm. The quantification of the ABTS radical scavenging activity of the samples was carried out by comparing with a standard calibration curve of Trolox, a water-soluble vitamin E analog. The antioxidant capacity of the sample was evaluated in comparison to Trolox. This method exhibits a reliable measure of the sample's ability to neutralize the $ABTS^+$ radical and indicates its overall antioxidant efficiency.

Assays for Total Phenolic and Flavonoid Contents: The total phenolic content was determined by employing the methods given in the literature with some modification. Sample solution (0.25 mL) was mixed with diluted Folin–Ciocalteu reagent (1 mL, 1:9, v/v) and shaken vigorously. After 3 min, Na2CO3 solution (0.75 mL, 1%) was added and the sample absorbance was read at 760 nm after a 2 h incubation at room temperature. The total phenolic content was expressed as milligrams of gallic acid equivalents (mg GAE/g extract).

The total flavonoid content was determined using the AlCl3 method. Briefly, sample solution (1 mL) was mixed with the same volume of aluminum trichloride (2%) in methanol. Similarly, a blank was prepared by adding sample solution (1 mL) to methanol (1 mL) without AlCl3. The sample and blank absorbances were read at 415 nm after a 10 min incubation at room temperature. The absorbance of the blank was subtracted from that of the sample. Rutin was used as a reference standard and the total flavonoid content was expressed as milligrams of rutin equivalents (mg RE/g extract).

HPLC analysis

The formulation was subjected to a reversed-phase HPLC-UV analysis, in gradient elu-tion mode, to quantitatively determine the phenolic composition. The HPLC ap-paratus consisted of a two PU-2080 PLUS chromatographic pump, a DG-2080-54-line degasser, a mix-2080-32 mixer, UV, an AS-2057 PLUS autosampler and a CO-2060 PLUS column thermostat (all from Jasco, Tokyo, Japan). ChromNAV2 Chromatog-raphy software was used for integration.

The separation was conducted within 60 min of the chromatographic run, starting from the following separation conditions: 97 % water with 0.1 % formic acid, and 3 % methanol with

0.1 % formic acid. The details about gradient are listed in Table S1. The separation was performed on an Infinity lab Poroshell 120-SB reverse phase column (C18, 150 × 4.6 mm i.d., 2.7 µm; Agilent, Santa Clara, CA, USA). Column temperature was set at 30 °C. Quantitative determination of phenolic compounds was performed via UV detector at 254 nm. The injection volume was 5 µL. Quantification was done through 7-point calibration curves, with linearity coefficients (R2) > 0.999, in the con-centration range 2–140 µg/mL. The final concentration of the extract was 40 mg/mL before injecting in HPLC system.

TIME (min)	COMPOSITION A% (Water + Formic acid 0.1%)	COMPOSITION B% (Methanol + Formic acid 0.1%)	FLOW (mL/min)
1	97	3	0.6
5	77	23	0.6
12	73	27	0.6
18	57	43	0.6
25	52	48	0.6
32	50	50	0.6
34	50	50	0.6
37	35	65	0.6
40	5	95	0.6
47	10	90	0.6
48	10	90	0.6

Table S1. Gradient elution condition for chromatographic determination (HPLC-UV) analysis of phenolic compounds. UV detector was set at 254 nm.

Allelopathy assay

An experiment using Petri dishes was designed to investigate the phytotoxic effects of the tested formulation within a concentration range of 0.62 to 10 mg/mL. The seeds of Cichorium intybus (CI), Dichondra repens (DR), and Raphanus sativus (RS), which are commercially available dicotyledons, were chosen due to their rapid germination and high sensitivity to external agents. These traits make them reliable indicators for detecting subtle inhibitory effects. By incorporating these species, the study aimed to comprehensively evaluate the formulation's potential to impact different plant species and determine whether it exerts broad-spectrum or selective phytotoxicity.

The assay was conducted in 90 mm Petri dishes lined with double-layered filter paper discs, which were soaked with 3 mL of the extract at varying concentrations. Distilled water was used as the negative control. The seeds were sterilized by soaking in a diluted bleach solution (NaClO:dH2O, 1:9) for 10 minutes, followed by thorough rinsing with sterile distilled water to remove residual bleach. Ten seeds from each plant type were placed on the filter paper discs, with the Petri dishes divided into three sections to separate the seed varieties. The dishes were sealed with parafilm to maintain a controlled environment and incubated in darkness at a temperature of 25±2°C for 96 hours.

Germination was identified by the appearance of a radicle extending at least 2 mm and showing natural geotropic curvature, while seeds that only swelled without full germination were excluded. The length of the seedlings was measured and classified into three categories: low (<0.4 cm), medium (0.5–0.9 cm), or high (>1 cm). After 96 hours, the germination rate and seedling lengths were recorded. Results were reported as Germination Percentage (GP) and Seedling Length (SL), calculated as the average seedling length relative to the control group.

Brine shrimp toxicity bioassay

Cysts of *Artemia salina* L. were incubated in oxygenated artificial seawater (1 g cysts/L) with a salinity of 32 g/L to facilitate hatching. After 24 hours, the toxicity evaluation was performed using the Brine Shrimp Lethality Assay (BSLA) as outlined by Meyer et al. [1] and McLaughlin et al. [2], employing freshly hatched nauplii. Triplicate tests were conducted for each concentration, ranging from 0.625 to 10 mg/mL. Each glass tube contained 5 mL of artificial seawater and 10 nauplii. After 24 hours of exposure, the number of surviving nauplii was recorded, and the mortality rate was calculated using the formula: ((T−S)/T)×100, where T represents the total number of larvae introduced, and S denotes the number of surviving nauplii. The median lethal concentration (LC50) was determined using GraphPad software. The test was considered valid if mortality in the control group did not exceed 10%.

Daphnia magna cardiotoxicity assay

For each experimental group, three non-pregnant *Daphnia magna* specimens were selected and allocated to individual wells of a 6-well plate, with three specimens per well. One well contained spring water as the control group, while another contained the formulation at a concentration of 6.866 mg/mL, equivalent to the LC_{50} value determined in the previous BSLA experiment. The specimens were exposed to the assigned treatments for 15 minutes at room temperature. After the exposure period, each *D. magna* specimen was individually transferred to a microscope slide containing a 50 µL droplet of the tested formulation, and their heartbeat rates were observed and recorded under a microscope for 15 seconds. To assess the cardiotoxic response, the specimens were subsequently exposed to a 10% ethanol solution for 2 minutes.

All observations were conducted in triplicate, and the experiment was repeated three times for consistency. The results, specifically the decrease in heart rate, were compared across the untreated group (negative control), the ethanol-treated group (positive control), and the group treated with the tested formulation.

Cell culture

The cells were cultured in DMEM (Dulbecco's Modified Eagle Medium) supplemented with 20% fetal bovine serum (FBS) and 1% penicillin-streptomycin, in a humidified atmosphere of 5% CO_2 at

37°C. Cells were routinely monitored under an inverted microscope to ensure proper morphology and confluence. Upon reaching approximately 70% confluence, cells were detached using 0.05% trypsin-EDTA, passaged, and seeded at appropriate densities for subsequent assays.

Determination of Cell Viability

The MTT assay was used to evaluate the effect of the tested formulation on the viability of C2C12 cells. This assay, widely employed to assess cell viability and cytotoxicity, relies on the reduction of MTT (3-(4,5-dimethylthiazol-2-yl)-2,5-diphenyltetrazolium bromide) by mitochondrial dehydrogenases in viable cells to produce insoluble formazan crystals. The purple formazan, dissolved in DMSO and quantified spectrophotometrically at 570 nm.

To assess cell viability under baseline conditions, C2C12 cells were seeded in 96-well plates at a density of 5×10^3 cells/well and allowed to adhere for 24 hours. The formulation was then administered at final concentrations of 200, 500 and 1000 μg/mL for 24 hours. After treatment, MTT solution (5 mg/mL) was added to each well and incubated for 3 hours at 37°C. Formazan crystals were solubilized in DMSO, and absorbance was measured at 570 nm using a microplate reader. Control wells containing medium without cells were used as blanks, and cell viability was calculated relative to untreated control wells.

For evaluating the effect of the formulation under H_2O_2-induced oxidative stress, the same experimental setup was used. Following the 24-hour treatment with the formulation, H_2O_2 was added at final concentrations of 500 μM for 3 hours to induce oxidative stress. After H_2O_2 exposure, the medium was replaced with MTT solution (5 mg/mL) and incubated for 3 hours at 37°C. Formazan crystals were dissolved in DMSO, and absorbance was measured at 570 nm. Control wells containing medium without cells were used as blanks, and cell viability was determined relative to untreated control wells.

Gene expression analysis

Total RNA was extracted from C2C12 cells using TRI reagent (Sigma-Aldrich, St. Louis, MO, USA), according to the manufacturer's protocol, and reverse transcribed using High Capacity cDNA Reverse Transcription Kit (ThermoFischer Scientific, Waltman, Massachusetts, USA). Specifically, 1 μg of total RNA extracted from each sample in a 20 μl reaction volume was reverse transcribed using High Capacity cDNA Reverse Transcription Kit (Applied Biosystems, Foster City, CA, USA). Reactions were incubated in a 2720 Thermal Cycler (Applied Biosystems, Foster City, CA, USA) initially at 25 °C for 10 min, then at 37 °C for 120 min, and finally at 85 °C for 5 s. Gene expression was determined by quantitative real-time PCR using TaqMan probe-based chemistry. PCR primers and TaqMan probes were purchased from Thermo Fisher Scientific Inc. The Assays-on-Demand Gene Expression Products used for gene expression evaluations in the mouse cortex specimens were: Mm00446190_m1 for IL-6 gene, Mm04336676_m1 for PRLR, Mm00433149_m1 for ESR1, Mm00443258_m1 for TNFα, Mm00441908_m1 for RANKL, Mm0607939_s1 for β-actin gene. β-actin was used as the housekeeping gene. The elaboration of data was conducted with the Sequence Detection System (SDS) software version 2.3 (ThermoFischer Scientific). Relative quantification of gene expression was performed by the comparative $2^{-\Delta\Delta Ct}$ method.

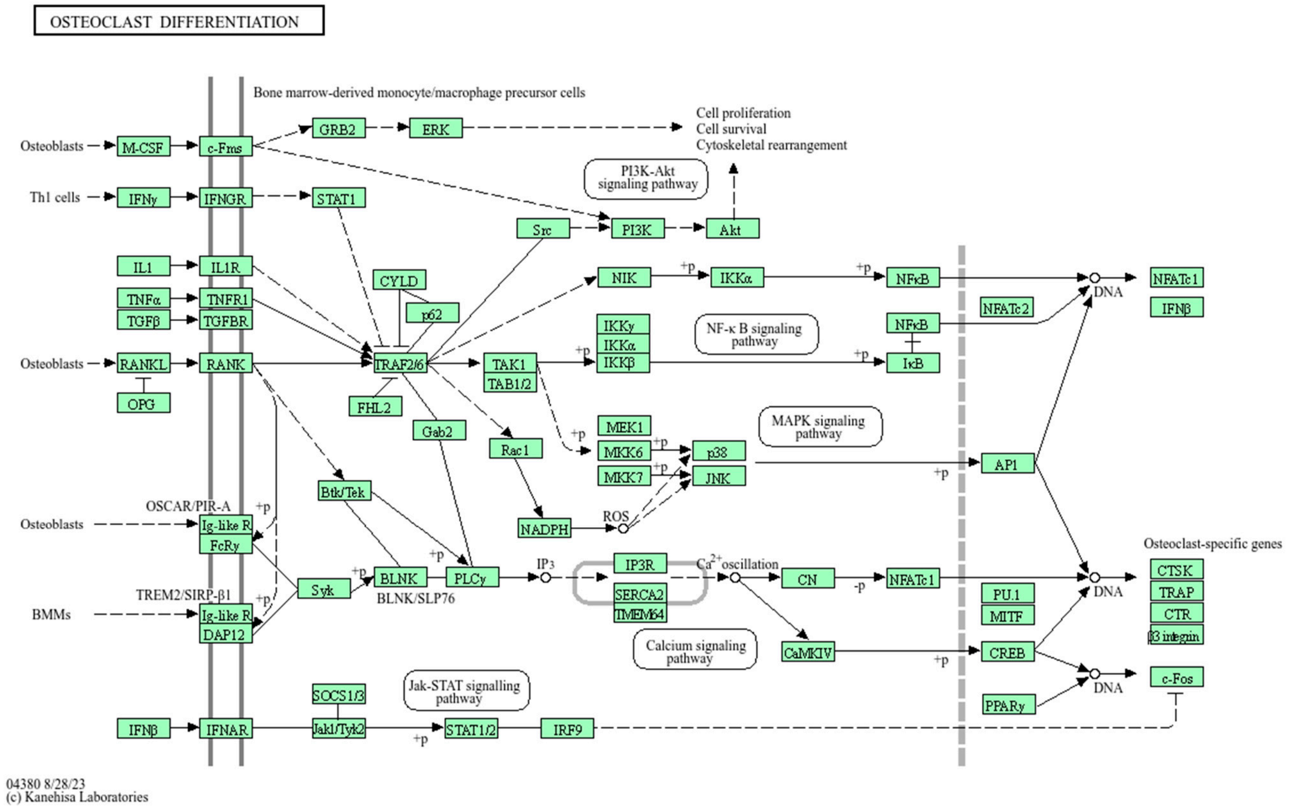

Figure S1. Osteoclast differentiation pathway (hsa04380; https://www.kegg.jp/kegg-bin/show_pathway?hsa04380).

References

1. Meyer, B.; Ferrigni, N.; Putnam, J.; Jacobsen, L.; Nichols, D.; McLaughlin, J. L., Brine shrimp: a convenient general bioassay for active plant constituents. Planta medica 1982, 45, (05), 31-34.

2. McLaughlin, J. L.; Rogers, L. L.; Anderson, J. E., The use of biological assays to evaluate botanicals. Drug information journal 1998, 32, (2), 513-524.
